# Preferential Solvation of Zwitterionic Benzo-[f]-Quinolinium Ylids in Binary Solvent Mixtures: Spectral Study and Quantum Chemical Calculations

**DOI:** 10.3390/molecules31020290

**Published:** 2026-01-13

**Authors:** Mihaela Iuliana Avadanei, Ovidiu Gabriel Avadanei, Dana Ortansa Dorohoi

**Affiliations:** 1 Petru Poni Institute of Macromolecular Chemistry, 41A Gr. Ghica Voda Alley, 700487 Iasi, Romania; 2 Faculty of Physics, Alexandru Ioan Cuza University, 11 Carol I Blvd, 700506 Iasi, Romania; minu@uaic.ro (O.G.A.); ddorohoi@uaic.ro (D.O.D.)

**Keywords:** negative solvatochromism, binary mixtures, preferential solvation, zwitterionic ylids, intermolecular hydrogen bonding

## Abstract

Three derivatives of benzo-[f]-quinolinium ylids, which all underwent an intermolecular charge transfer process, were used as solvatochromic indicators to study the specific solvent–solute interactions in binary mixtures of protic–aprotic solvents with different molar ratios. The microenvironment around the solute molecules was observed via electronic absorption spectroscopy and was analyzed by employing solvation models and quantum chemical calculations. The spectral analysis suggested that the solute was preferentially solvated by the polar protic solvent, indicating a lack of synergy between the two solvents. The solvation microsphere was progressively occupied by the protic solvent, on the basis of specific solute–solvent interactions. By modeling the 1:2 (solute-coordinating solvent) complexes with explicit solvents, the binding energy for complex formation was estimated.

## 1. Introduction

Binary solvent mixtures provide the ideal medium to investigate the competition between several types of solvation phenomena. As a series of biological processes and chemical reactions in living systems takes place in binary solvents, the solvation of relevant molecular probes can be viewed as a molecular recognition tool. The close relationship between the microscopic (hydrogen-donating and -accepting abilities and polarizability) and macroscopic (polarity, viscosity, refractive index, and permittivity) properties of the solvent often governs the composition of the first solvation sphere of a dipolar solute, with serious consequences on its energetics. From a spectral point of view, the shifts in the electronic absorption or fluorescence bands are accurate indicators of the polarity and coordinating properties of the two solvents and their effect on the solute. This is because the first solvation shell is a different entity from the rest of the solution, consisting of a unique arrangement of the two solvents around the solute.

Within this context, dipolar/zwitterionic probes with separated charges are even more interesting because of their intermolecular charge transfer phenomenon (ICT). It is worth noting that zwitterionic dyes with pronounced negative solvatochromism, like Reichardt’s betaine dye 30 (2,6-diphenyl-4-(2,4,6-triphenylpyridinium-1-yl)-phenolate), have been used to determine the *E_T_*(30) and $E_{T}^{N}$ polarity parameters of solvents [1,2,3]. The scientific community maintains an interest in the negative solvatochromism of zwitterionic dyes because the polarity scale of the solvents and the measurement of their properties (Kamlett–Abboud–Taft π*, α, and β parameters) are continuously investigated and are being continually updated. Additionally, the solvatochromism of such probes is an essential tool for analyzing the (micro)polarity of microheterogeneous systems such as micelles, microemulsions, or phospholipid bilayers [2,4].

As is the case in the family of Reichardt’s betaine B30 or in merocyanines, the negative solvatochromism of zwitterionic dyes is dependent on a succession of chemical changes induced by the solvent polarity, the electrophilic or nucleophilic character of the solvent, and the coordinating properties of both the solvent and solute [5,6]. Their behavior in solvent mixtures is more complicated, as each solvent has its own characteristics and many binary systems act like non-ideal solvents. The local composition of the first solvation shell dictates the overall solvation behavior. The preferential solvation is observed when the solute establishes close and strong intermolecular interactions with one of the solvents. In synergic systems, the two solvents are more attracted to each other than to the solute, forming an intersolvent complex that solvates the solute.

**Figure 1:**
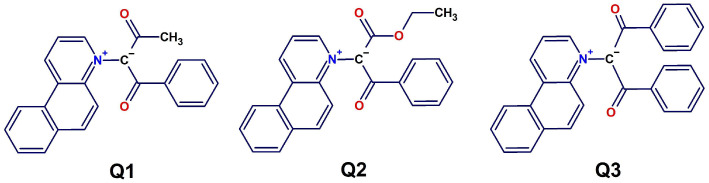
Chemical structures of the zwitterionic probes used in this study.

In a recent study, we reported the negative solvatochromism in pure solvents of three benzo-[f]-quinolinium methylide derivatives: benzo-[f]-quinolinium acetyl benzoyl methylid (Q**1**), benzo-[f]-quinolinium carbethoxy benzoyl methylid (Q**2**), and benzo-[f]-quinolinium dibenzoyl methylid (Q**3**) (Figure 1) [7]. Quinolinium ylid derivatives are very good H-bond acceptors, and Platts and Howard [8] and Rozas et al. [9] have shown that hydrogen bonding, even at the carbanion site with weak HB donors, may lead to stable H-bonded complexes.

The charge-separated ground state of Q**1**–Q**3** can be explained by its special chemistry: a polarizable polyheterocyclic structure, allowing the study of dispersion interactions; a permanent dipole moment; and a sterically shielded positive nitrogen atom. The longest absorption band appears at around 488 nm in inert solvents and exhibits a bathochromic shift of about 80 nm in alcohol [7]. Electronic excitation into the first excited state is accompanied by a reduction in the dipole moment due to the charge transfer from the carbanion to the positive nitrogen atom. Based on the HB acceptor and electron acceptor/donor characteristics, we have attempted to use Q**1**–Q**3** as indicators and targets to investigate solute–solvent and solvent–solvent interactions in binary mixtures.

Four sets of solvent combinations were prepared from one inert or weak HB donor/acceptor solvent (benzene, 1,2-dichloroethane, chloroform, and N,N-dimethylformamide) and one strong HB donor/acceptor solvent (propanoic acid, methanol, octanol, and propane-1,3-diol). We hypothesized that Q**1**–Q**3** would easily recognize the dipolar protic solvent via specific interactions, thereby shaping the solvation microsphere in a specific pattern. The role of the solvents in the solvatochromic responses was analyzed in relation to the composition of the solvation microsphere by using the preferential/synergic solvation models of Bagchi–Chatterjee [10,11], Bosch–Rosés [12,13], and the statistical cell model for ternary solutions [14,15,16,17]. Finally, the spectrochromic behavior was analyzed via quantum chemical calculations with DFT methods.

## 2. Results and Discussion

### 2.1. Solvatocromism in Binary Mixtures

**Figure 2:**
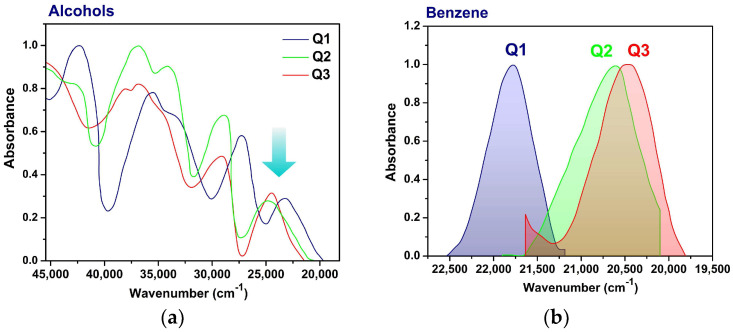
(**a**) Experimental electronic absorption spectra in methanol, with the ICT band highlighted by the arrow; (**b**) the position of the ICT band in benzene.

In our previous report, detailed geometry analysis revealed that the rotation around the single C^−^–N^+^ bond gives the molecules a highly twisted conformation, and this directly affects the profile of the UV–Vis spectra [7]. Intramolecular charge transfer (ICT) occurs from the carbanion to the polyheterocycle and is highly sensitive to the properties of the surrounding medium [7]. For example, Figure 2a,b show the redshift of the ICT band of Q**1**–Q**3** from a methanol solution to a benzene solution, indicating a clear connection to the hydrogen-bonding properties of methanol. Table 1 presents the solvatochromic parameters of the solvents used in this study and the absorption maxima of Q**1**–Q**3** in the pure solvents. From polar to non-polar solvents, the bathochromic shift increased from Q**1** to Q**3**, and there was a shift to lower energies with an increasing alkyl chain of alkanols, showing the hydrophobic effect of long alcohols.

For the solvents listed in Table 1, the variation in the maximum ICT absorption with the solvatochromic parameters π*, α, and β of neat solvents is described in terms of the Kamlet–Abboud–Taft approach, via the relationships (1)–(3) [18,19]:

ν(Q1) = 21068.94 + 944.26·π* + 1762.47·α + 602.29·β  (R^2^ = 0.95) (1)

ν(Q2) = 20369.25 + 890.18·π* + 2366.13·α + 1575.29·β  (R^2^ = 0.92), (2)

ν(Q3) = 19887.07 + 1202.02·π* + 2740.73·α + 1579.44·β  (R^2^ = 0.95) (3)

Equations (1)–(3) led to the conclusion that the polarizability had the least contribution to the negative solvatochromism of Q**1**–Q**3**, and the most important factor was the hydrogen bond–donating property of the solvents.

Further, we decided to investigate how the composition and the intermolecular interactions of the first solvation sphere of Q**1**–Q**3** change when a dipolar protic solvent is gradually included within. We selected four types of binary mixtures, combining a co-solvent with strong hydrogen donor and acceptor character (HBD, HBA) (solvent S1) with a solvent with very poor or non-coordinating properties (solvent S2). The solutions were methanol + benzene (I), propane-1,3-diol + N,N-dimethylformamide (II), 1-octanol + 1,2-dichloroethane (III), and propanoic acid + chloroform (IV). The experimental data for the maximum ICT band in every binary mixture are provided in Tables S1–S9 in the Supplementary Materials. The molar transition energies (in kcal/mol) of the charge transfer band of Q**1**–Q**3** in the mixtures (I)–(IV) were calculated according to the formula $E_{T}(kcal/mol)=28,590/\lambda_{max}$ [5].

**Figure 3:**
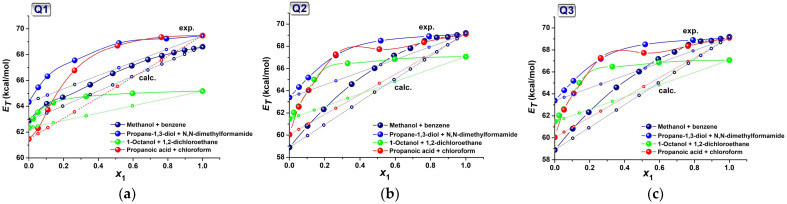
Variation in the molar transition energy with mole fractions of the polar solvent S1, *x*_1_, for (**a**) Q**1**; (**b**) Q**2**; (**c**) Q**3**. The dotted lines are the ideal case for no preferential solvation of Q**1**–Q**3**, and connect the calculated values of *E_T_* according to Equation (4). The solid lines are a visual guide.

The *E_T_* values were plotted against the molar fraction of the polar solvent S1, *x*_1_, and are shown in Figure 3. The continuous increase in the transition energy with the progressive addition of the alkanol confirms the stabilization of the zwitterionic ground state and the slight destabilization of the first excited state. The higher alcohols, such as octanol, produced a modest blue shift in the ICT band, and the transition energy was much lower than that for the other solvents. All graphs in Figure 3 are curvilinear (with solid lines and solid points), and departed from the ideal lines (open symbols) with a quantity reflecting the influence of the local composition of the solvents on the solute molecule. The molar energy transition of the solute in an ideal binary mixture, where the solvation shell is equally filled with both solvents, is described by the simple additivity law (4) [20]:

(4) $$E_{T}=E_{T1}\cdot x_{1}+E_{T2}\cdot x_{2}$$

The deviation from Equation (4) indicates that the composition of the microsolvation sphere was largely different from the bulk mixture. Figure 3 shows that these differences exist and are most probably due to interactions between the solute and the polar solvent S1. The behavior of the methanol + benzene mixture is quite peculiar, as the extent of preferential solvation is the lowest in all cases and is the closest to the ideal case. In this solution, hydrogen-bonded complexes between methanol and benzene cannot appear; otherwise, the polarity of the mixture would be higher than that of the individual solvents [12,13]. The *E_T_* vs. *x*_MeOH_ variation in Figure 3a suggests a relative equilibrium between methanol and benzene in the occupancy of the solvation microsphere of the solute. The deviation from additivity is the largest for Q**2** and Q**3** at methanol molar fractions above 0.6. This can be explained by the self-associating ability of methanol, which preferentially associates with other methanol molecules over the solute [21,22,23], forming clusters and linear chains that interact weakly with the benzene molecules. This competition is not observed for halogenated solutions (III) and (IV). Therefore, the preferential solvation of Q**1**–Q**3** by the polar solvent S1 occurred in the S2-rich regions.

### 2.2. General Analysis of the Preferential Solvation in Binary Mixtures

The ICT transition energy of Q**1**–Q**3** in binary mixtures, *E_T_*, can be mathematically described in an expression containing the local molar fractions of the two solvents, $x_{1}^{s}$ and $x_{2}^{s}$, in the first solvation shell, in which the preferential solvation of Q**1**–Q**3** manifests itself [10,11]:

(5) $$E_{T}=E_{T1}{\cdot x}_{1}^{s}+E_{T2}{\cdot x}_{2}^{s}$$

(6) $$x_{1}^{s}=\frac{E_{T}-E_{T2}}{E_{T1}-E_{T2}}$$

According to Bagchi and Chatterjee [10,11], the deviation from the additivity law (4) measures the extent of the preferential solvation and is given by the difference $\delta_{s}$ in molar fractions of solvent S1 between the microsolvation sphere and the bulk composition, according to

(7) $$\delta_{s}=x_{1}^{s}-x_{1}$$

The graphical representation of $x_{1}^{s}$ as a function of the molar fraction of S1 in the bulk mixture, as presented in Figure S1, mirrors the behavior of the transition energy of the ICT band (Figure 3) with *x*_1_. The deviation from the ideal binary mixtures indicates a preference for the polar solvent S1, a trend that is smooth in methanol + benzene and is more pronounced in 1-octanol + 1,2-dichloroethane.

**Figure 4:**
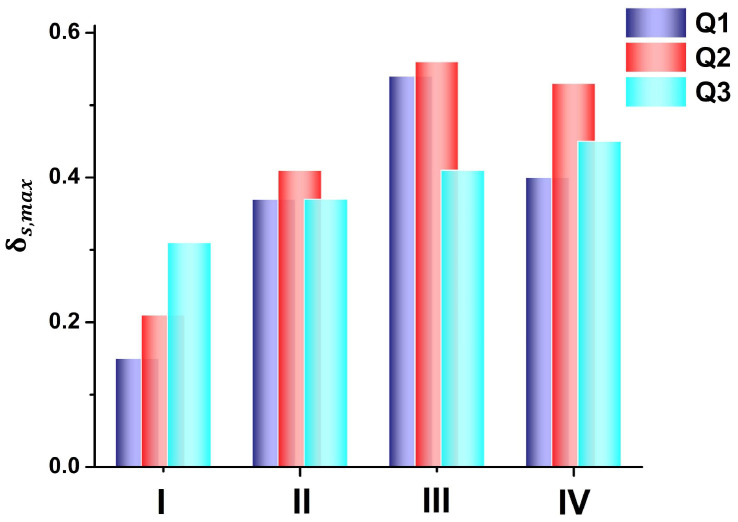
Maximum deviation from the ideal solvation of the mole fraction of the polar solvent S1, $\delta_{s},max$, in binary mixtures of I—methanol + benzene; II—propane-1,3-diol + N,N-dimethylformamide; III—1-octanol + 1,2-dichloroethane; and IV—propanoic acid + chloroform.

The highest value of $\delta_{s}$ in the mixtures is illustrated in Figure 4. Following the dependence of *E_T_* and $x_{1}^{s}$ on $x_{1}$, the smallest $\delta_{s},max$ value is 0.2 on average and is found in the methanol + benzene solution, which is the least H-bonded of all four systems. The highest preferential solvation is observed in 1-octanol + 1,2-dichloroethane, where $\delta_{s},max$ ≈ 0.5. For Q**1**, $\delta_{s},max$ ≈ 0.5 corresponds to an *E_T_* (ideal–experimental) difference of around 0.5 kcal/mol in methanol + benzene and around 3 kcal/mol in propanoic acid + chloroform. In the binary solutions (III) and (IV), the energy differences *E_T_* (ideal–experimental) at $\delta_{s},max$ ≈ 0.5 are between 3 and 4.75 kcal/mol. They are observed at a mole fraction of 1-octanol or propanoic acid of ≈0.3. The preferential solvation of Q**1**–Q**3** by 1-octanol (in solution III) or by propanoic acid (in solution IV) only partially follows the dielectric enrichment principle of the Suppan model, where the most dipolar solvent fills the solvation shell of the dipolar solute [24,25]. While this principle is valid for the methanol + benzene solution, it cannot be completely correct for 1-octanol and 1,2-dichloroethane (solution III), which have the same dielectric permittivity. The deviation from additivity in this case comes from the specific interactions between the solute and solvents and between the two solvents.

**Figure 5:**
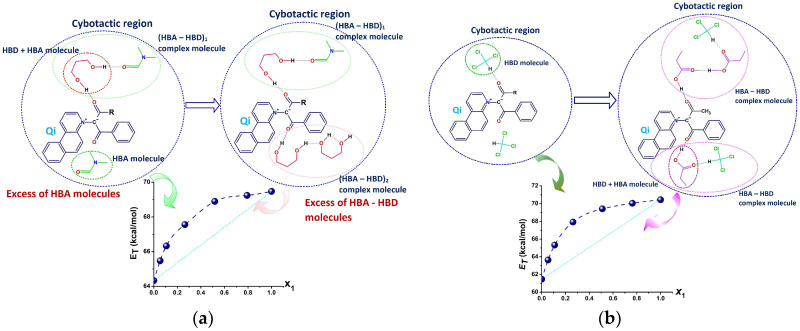
Cybotactic regions of Q**1**–Q**3** in (**a**) propane-1,3-diol + N,N-dimethylformamide; and (**b**) propanoic acid + chloroform. The dashed lines illustrate the specific interactions between the solute and the two solvent types, with hydrogen bond acceptor (HBA) and hydrogen bond donor (HBD) properties.

Knowing the coordinating properties of the solvents and the sensitive functional groups of Q**1**–Q**3**, Figure 5 presents the most probable solvent–solvent and solute–solvent interactions in two of the four binary mixtures: propane-1,3-diol + N,N-dimethylformamide (Figure 5a) and propanoic acid + chloroform (Figure 5b).

Unlike propanoic acid, which can self-associate into dimers, N,N-dimethylformamide has a low degree of interaction between its molecules [26], because it is a very poor H-bond donor. Formyl oxygen is a very good acceptor, and in mixed solvents, N,N-dimethylformamide molecules prefer to associate with the co-solvent. Intermolecular interactions between Q**1**–Q**3** and chloroform are expected to be at a low extent, as judged from the position of the ICT band. As the λ_max_ in propanoic acid is the lowest of all four binary solvents used, it appears that the interactions of Q**1**–Q**3** with the acid are the strongest of all studied. Therefore, Figure 5b cannot fully capture the complexity of these interactions.

### 2.3. Preferential Solvation Analyzed Through the Bosch–Rosés Model

The UV–Vis data and the plots of *E_T_* vs. molar fraction for the polar co-solvent S1 suggest a preferential solvation of Q**1**–Q**3** by S1, and that a potential interactive complex solvent has been formed in some binary mixtures. As we have pointed out above, the methanol + benzene complex appears to have a weak interaction energy, and that can be explained by the experimental and theoretical studies on benzene–methanol clusters by Pribble et al. [22] and Matisz et al. [23]. The preferential solvation model is based on two exchange processes in the solvation sphere of Q**1**–Q**3** molecules [12,13]:

(8) $$Q_{i}(S2)+S1\;⇌Q_{i}(S1)+S2$$

(9) $$Q_{i}(S2)+S2{S1}_{2}⇌Q_{i}{(S12}_{2})+S2$$

where Qi collectively designates the Q**1**–Q**3** molecules, solvated by S1 ($Q_{i}(S1)$) and S2 ($Q_{i}(S2)$) and by an intermolecular complex between S1 and S2 ($Q_{i}(S12)$). The specific and non-specific interactions between the solute and the solvents in the solvation microsphere are expressed in the Bosch–Rosés model [13] using two parameters specific to the one-to-one S12 complex, so that the relation (5) becomes

(10) $$E_{T}=E_{T1}{\cdot x}_{1}^{s}+E_{T2}{\cdot x}_{2}^{s}+E_{T12}{\cdot x}_{12}^{s}$$

where $x_{12}^{s}$ is the molar fraction of the S12 complex in the cybotactic region. *E_T_*_12_ is the ICT transition energy corresponding to the $x_{12}^{s}$ fraction. The relationship between the molar fractions of S1 and S2 in the bulk mixture and in the first solvation shell is linear and is given in Equation (10):

(11) $$x_{1}+x_{2}=x_{1}^{s}+x_{2}^{s}+x_{12}^{s}=1$$

The preferential solvation of Q**1**–Q**3** by the co-solvent S2 or by the solvent complex S12, with reference to the non-polar solvent S1, is described by two process parameters, $f_{2/1}$ and $f_{12/1}$ (Equations (12) and (13)). The solvating ability of the mixed S12 relative to S2 is given by $f_{12/2}$ (Equation (14)).

(12) $$f_{2/1}=\frac{x_{2}^{s}/x_{1}^{s}}{{\left(x_{2}/x_{1}\right)}^{2}}$$

(13) $$f_{12/1}=\frac{x_{12}^{s}/x_{1}^{s}}{x_{2}/x_{1}}$$

(14) $$f_{12/2}=\frac{f_{12/1}}{f_{2/1}}$$

The ICT transition energy of Q**1**–Q**3** when solvated by the S12 pair of solvents, *E_T_*_12_, is then obtained from the relation (15), while the local molar fractions $x_{1}^{s}$, $x_{2}^{s}$ and $x_{12}^{s}$ are calculated from relations (16)–(18):

(15) $$E_{T}=\frac{E_{T1}\cdot {\left(x_{1}\right)}^{2}+E_{T2}\cdot f_{2/1}{\left(1-x_{1}\right)}^{2}+E_{T12}\cdot f_{12/1}\cdot x_{1}\cdot \left(1-x_{1}\right)}{{{(x}_{1})}^{2}+f_{2/1}{\left(1-x_{1}\right)}^{2}+f_{12/1}\cdot x_{1}\cdot \left(1-x_{1}\right)}$$

(16) $$x_{1}^{s}=\frac{{\left(x_{1}\right)}^{2}}{{\left(x_{1}\right)}^{2}+f_{2/1}{\left({1-x}_{1}\right)}^{2}+f_{12/1}x_{1}(1-x_{1})}$$

(17) $$x_{2}^{s}=\frac{f_{2/1}{\left({1-x}_{1}\right)}^{2}}{{\left(x_{1}\right)}^{2}+f_{2/1}{\left({1-x}_{1}\right)}^{2}+f_{12/1}x_{1}(1-x_{1})}$$

(18) $$x_{12}^{s}=\frac{f_{12/1}x_{1}(1-x_{1})}{{\left(x_{1}\right)}^{2}+f_{2/1}{\left({1-x}_{1}\right)}^{2}+f_{12/1}x_{1}(1-x_{1})}$$

The parameters *E_T_*_12_, *f*_2/1_, and *f*_12/1_ were determined through the nonlinear regression of Equation (15) and are listed in Table 2. Several remarks can be made accordingly:

(i) The values of *f*_2/1_ are lower than for unity, except for the methanol + benzene mixture, meaning that Q**1**–Q**3** are more solvated by the polar solvent S1; *f*_2/1_ is very small for 1-octanol + 1,2-dichloroethane, which shows the occupancy of the cybotactic region by the alkanol molecules.
(ii) The values of *f*_12/1_ are higher than those of *f*_2/1_ for any mixture under study, which suggests that the solute molecules are preferentially solvated by the solvent intercomplex S12 in reference to the polar solvent S1; this effect is important for propane-1,3-diol + N,N-dimethylformamide mixtures and is irrelevant for the propionic acid + chloroform solution, confirming that propionic acid is the preferred solvent.
(iii) The *f*_12/2_ parameter, measuring the tendency of Q**1**–Q**3** to be solvated by the S12 complex rather than the aprotic solvent S2, is higher than unity, showing that S12 is preferred over the non-polar solvent.
(iv) Within this context, the cybotactic region of Q1 was mainly composed of methanol–benzene, propane-1,3-diol–N,N-dimethylformamide mixtures, or 1-octanol–1,2-dichloroethane complexes; Q**2** and Q**3** show a lower preference for solvent complexes.
(v) The values of *f*_2/1_ are only higher than 1 in the methanol + benzene mixture, which could be interpreted as a preference for benzene over methanol; however, for *f*_12/1_ > *f*_2/1_, the functional groups of Q**1**–Q**3** can be selectively solvated by one of the two solvents; therefore, the solvation microsphere is not uniformly filled with solvent molecules, but they are selectively arranged around the HBA groups of the solute.
(vi) For the combinations with alcohols, *f*_2/1_ decreases as the chain and the hydrophobicity of the alcohol increases; the lowest values are for 1-octanol + 1,2-dichloroethane, due to the very poor coordinating ability and high polarizability of 1,2-dichloroethane.
(vii) The *E_T_*_12_ values are an intermediary between *E_T_*_1_ and *E_T_*_2_ for any mixture; for propanoic acid + chloroform, *E_T_*_12_ ≈ (*E_T_*_1_ + *E_T_*_2_)/2; and for the other three compositions, *E_T_*_12_ is closer to either *E_T_*_1_ or *E_T_*_2_, meaning that the intersolvent complex S12 is weak.

One may conclude that the studied binary solvent mixtures do not exhibit any synergism, as the *E_T_*_12_ values do not exceed those of the neat solvents. This means that the S12 complexes are less polar than the individual solvents. In conjunction with Figure 4 and Figure 5, it is obvious that the Q**1**–Q**3** molecules prefer the polar protic solvent in their microenvironment, because they are very good H-bonding acceptors.

A close analysis of the data in Table 2 reveals that among the three compounds, Q**3** is the most sensitive to the mixed composition of the solvation sphere, despite the identical substitution in the two branches at C^−^. The *E_T_*_12_ values are highest in the propane-1,3-diol + N,N-dimethylformamide mixture, followed by the propanoic acid + chloroform mixture. Again, it can be observed that propane-1,3-diol and N,N-dimethylformamide can form intermolecular complexes, contrasting with the low possibility for the other three mixtures under study. On the contrary, Q**1** appears to prefer the polar solvent over the S12 complex. Q**2** had a preference for S12 in the methanol + benzene and propane-1,3-diol + N,N-dimethylformamide mixture, which is the combinations with the highest probability to form solvent complexes.

**Figure 6:**
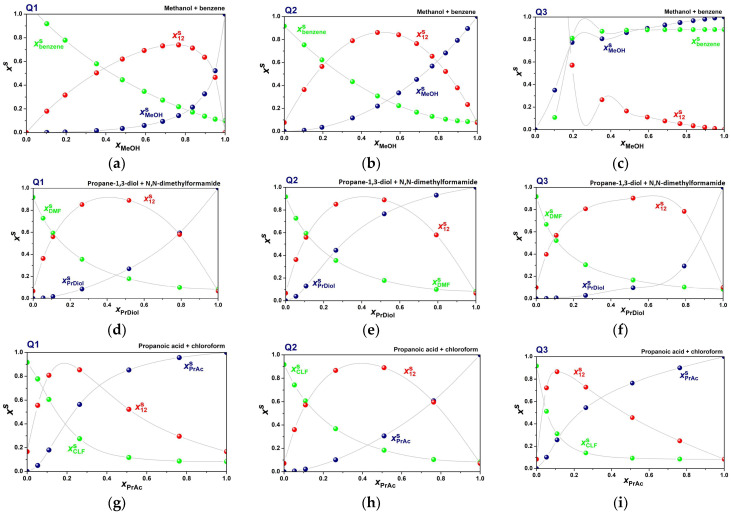

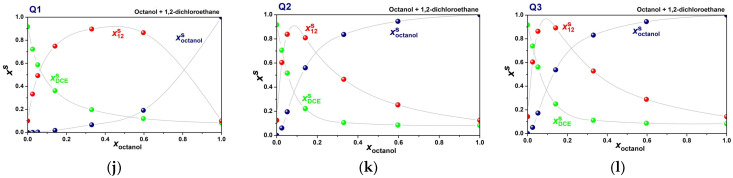
Local mole fractions around the molecules of Q**1**–Q**3** in the cybotactic region, calculated from Equations (16)–(18), in binary mixtures: (**a**–**c**) methanol + benzene; (**d**–**f**) propane-1,3-diol + N,N-dimethylformamide; (**g**–**i**) propanoic acid + chloroform; (**j**–**l**) 1-octanol + 1,2-dichloroethane. The lines are a visual guide.

The calculated mole fractions of S1, S2, and solvent complex S12 in the solvation microsphere are presented in Figure 6. With the exception of the propane-1,3-diol + N,N-dimethylformamide solution, differences are seen between all binary mixtures. This highlights the way in which the substitution at anionic carbon affects the interactions with the solvent: firstly, by the steric hindrance imposed by the benzene ring in Q**3** to the solvent molecules; and secondly, by the high polarizability of the same functional group.

### 2.4. Solvation of Q**1**–Q**3** Analyzed by Means of the Ternary Statistical Cell Model

In the statistical cell model of ternary solutions [14,15,16,27,28], S1 is a coordinating solvent, while S2 is almost inert. This condition marks a difference from the Bosch–Rosés model, which was developed for the interaction of solvents that possess high polarity and polarizability/dipolarizability. Therefore, the Bosch–Rosés model successfully analyzes synergic binary mixtures, while the statistical cell model is more appropriate for non-interacting binary mixtures. We have already seen that the S1–S2 interactions in our solutions I–IV and the polarity of the two solvents were not strong enough to construct a synergic (S12–solute) complex. The deviation from the ideality was solely attributed to the solvent–solute interactions, as Q**1**–Q**3** were preferentially solvated by the polar solvent S1.

Considering that the interaction energy between the solute molecule and its first solvation shell is higher compared with its superior solvation shells, the statistical cell model for ternary solutions takes into account microcanonical systems made by one solute molecule and its neighboring molecules from the first solvation shell. The solution is divided into independent microcanonical subsystems, with the rest of the solution as the reservoir. The solute’s concentration in the ternary solution determines the number of the considered microcanonical subsystems, and also the intensity in the electronic absorption spectrum. The strength of the interaction energy between the solute and the solvent molecules in liquids is of the same magnitude as the energy of the thermal motion. Therefore, the thermal agitation continuously changes the composition of the first solvation cage of the solute molecule, as the S1 and S2 solvent molecules compete with each other in order to occupy the most strongly favored place in the vicinity of the solute.

In short, the statistical cell model of ternary solutions introduces the interaction energy ω in the molecular pairs (solute–S1) and (solute–S2), noted as ω_1_ and ω_2_, respectively. These parameters are connected with the molar fraction of the polar solvent S1 from Equation (5), $x_{1}^{s}$, by a logarithmic relationship in the following form:

(19) $$ln\frac{x_{1}^{s}}{1-x_{1}^{s}}=ln\frac{x_{1}}{1-x_{1}}+\frac{\omega_{2}-\omega_{1}}{kT}$$

where *kT* = 4.11 × 10^21^ J, k = 1.380 × 10^−23^ J/K is the Boltzmann constant and *T* (K) is the temperature. The $\omega_{2}-\omega_{1}$ energy difference is calculated from the linear plot of $ln\frac{x_{1}^{s}}{1-x_{1}^{s}}$ vs. $ln\frac{x_{1}}{1-x_{1}}$. The spectral measurements assure good precision, but the restrictive hypothesis neglects the interactions between the two solvents and those of the solute with the superior solvation shells.

**Figure 7:**
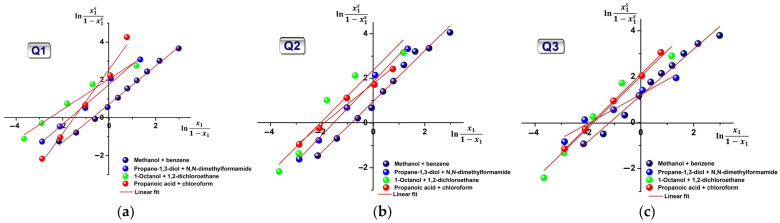
Plots of Equation (18) in all binary mixtures for (**a**) Q1; (**b**) Q2; and (**c**) Q3.

The corresponding graphical representations of $ln\frac{x_{1}^{s}}{1-x_{1}^{s}}$ vs. $ln\frac{x_{1}}{1-x_{1}}$ for Q**1**–Q**3** in all four binary solvents are illustrated in Figure 7. Table 3 lists the extracted data from the linear fitting. All plots are linear, confirming the validity of the chosen model for the binary mixtures under study. From Table 3, this results in the strongest interactions being established in halogenated ternary solutions, with the ω_2_ − ω_1_ difference in the order of 8–10 × 10^21^ J. As expected, the MeOH + benzene solution had the weakest interaction of all cases.

### 2.5. Quantum Chemical Calculations

The solvatochromic investigations suggest that the species in the ground state have different structures, depending on the coordinating abilities of the solvent. To test this observation, we used quantum chemical calculations to understand the effects of solvents on the Q**1**–Q**3** ground-state properties. We chose Q**1** as a model indicator. In the first stage, the solvent effect was accounted for using the implicit solvent formalism. The geometry of Q**1** was optimized in the gas phase and in the eight solvents that are components of the binary mixtures (Table S10).

The calculation of the frontier molecular orbitals of Q**1** and the estimation of the HOMO–LUMO energy gap are informative about negative solvatochromism and chemical reactivity [29,30]. The electrostatic potential maps illustrate the charge distribution and help identify potential sites that can act as H-bond acceptors. As one can observe in Figure S2, the electron density of HOMO is located around the carbanion in all cases, while the electron density of LUMO is moved to the polyheterocycle. The charge transfer process occurred in all solvents. The molecular electrostatic potential maps in the solvents, as compared to the gas phase, give a hint of the different conformations from solvation in one solvent to another. The red, green, and blue zones indicate the nucleophilic, neutral, and electrophilic regions of the molecule. The oxygen atoms from the carbonyl functions are highly electronegative, regardless of the solvent and the intermolecular bonding that they participate in. However, the electronegativity of oxygen atoms deepens from benzene to N,N-dimethylformamide, which is an indication of their high reactivity and of their position as acceptors of protons from the solvent. The polyheterocycle has a near-neutral potential, while the aromatic hydrogen atoms belong to the electrophilic zones.

**Figure 8:**
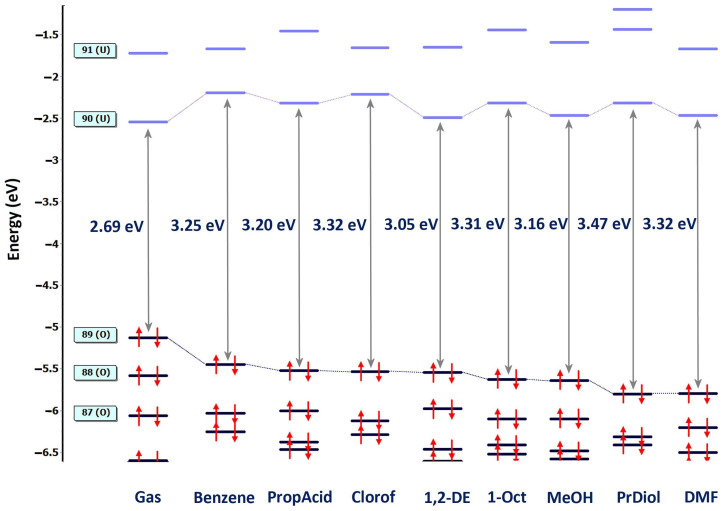
Diagram of energy levels of Q**1** in the eight solvents, with the number and occupancy of the relevant orbitals displayed. The abbreviations used are 1,2-DE for 1,2-dichloroethane, 1-Oct for 1-octanol, PrDiol for propane-1,3-diol, Clorof for chloroform, PropAcid for propanoic acid, and DMF for N,N-dimethylformamide (B3LYP/6-31G+(d,p)/IEF-PCM).

In terms of energy, Figure 8 shows that the increase in the solvent’s polarity led to the lowering of the HOMO level, from −5.12 eV in the gas phase to −5.80 eV in propane-1,3-diol and N,N-dimethylformamide. LUMO fluctuates around an average of −2.30 eV. The HOMO–LUMO energy gap decreased from benzene to propane-1,3-diol, with the latter showing the largest HOMO-LUMO distance (3.47 eV) and confirming negative solvatochromism. The Mulliken charge for N^+^ decreases from 0.9 e in benzene to 0.824 e in N,N-dimethylformamide.

The ionization potential, IP, and the electron affinity, EA, of Q**1** can be estimated from IP ≈ −E_HOMO_ and EA ≈ −E_LUMO_ [30]. The electronegativity is derived from the formula χ = (IP + EA)/2; the chemical potential is μ = −χ; the electrophilicity ω = μ^2^/2η [31]; and the hardness is given by η = ΔE = E_HOMO_ − E_LUMO_. The electronegativity, which is related to the chemical potential of the solute, and hardness, are essential in describing the chemical reactivity of a compound and in predicting the behavior towards reaction partners. The calculated values of these parameters are provided in Table 4.

The HOMO–LUMO gap, in descending order, is propane-1,3-diol > DMF = chloroform> 1-octanol > … > 1,2-dichloroethane; therefore, the reactivity of Q1 is highest in 1,2-dichloroethane and lowest in propane-1,3-diol. Simultaneously with the chemical potential μ, the electronegativity χ constantly increased from benzene to N,N-dimethylformamide, suggesting an increase in the energy of deformability of Q**1** in N,N-dimethylformamide and propane-1,3-diol.

**Figure 9:**
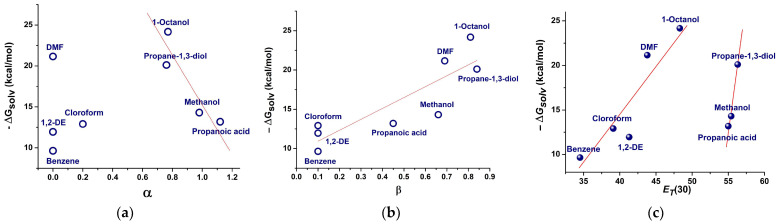
Relationships between the calculated Gibbs free energy of solvation, ${\Delta G}_{solv}$ of Q**1** and solvent parameters for the coordinating solvents: (**a**) the H-bond acidity; (**b**) H-bond basicity; and (**c**) the Dimroth polarity parameter, *E_T_*(30). The abbreviations used are 1,2-DE for 1,2-dichloroethane, and DMF for N,N-dimethylformamide. The red lines represent the linear fit of the calculated data points.

Next, the influence of the solvent was analyzed by calculating the Gibbs energy of solvation in implicit solvents, which represents the microscopic polarity around the solute. The plot of ${\Delta G}_{solv}$ against the acidity parameter α is shown in Figure 9a, and its plot against the basicity parameter β is shown in Figure 9b. Taking into account that the HB-accepting ability is zero for 1,2-dichloroethane, benzene, and N,N-dimethylformamide, the two clusters in Figure 9a were expected. As for the protic solvents, the ${\Delta G}_{solv}$ decreases in a linear relationship with the α parameter of the solvent Equation (20). There is a clear increasing trend of ${\Delta G}_{solv}$ with the HB donor abilities of the solvent, so the linear dependence (21) was deduced:

(20) $${\Delta G}_{solv}\left(kcal/mol\right)=42.37\left(\pm 4.88\right)-26.91\left(\pm 2.55\right)\cdot \alpha \;\;\;\;\;\;\;\;\;\;R^{2}=0.93$$

(21) $${\Delta G}_{solv}\left(kcal/mol\right)=9.54\left(\pm 0.95\right)+13.87\left(\pm 1.66\right)\cdot \beta \;\;\;\;\;\;\;\;\;\;R^{2}=0.88$$

The values in parentheses are the parameter standard errors.

The graphical representation of ${\Delta G}_{solv}$ vs. the polarity parameter *E_T_*(30) of the solvents [5], as shown in Figure 9c, yields the same separation into two clusters, as shown in Figure 9a. The linearity suggested by the data points can be expressed by Equation (22) for aprotic solvents and (23) for the protic ones:

(22) $${\Delta G}_{solv}\left(kcal/mol\right)=-30.83\;\left(\pm 1.98\right)+1.13\;\left(\pm 0.15\right)\cdot E_{T}(30)\;\;\;\;\;\;\;\;\;\;R^{2}=0.91$$

(23) $${\Delta G}_{solv}\left(kcal/mol\right)=-290.79\left(\pm 10.33\right)+5.51\left(\pm 0.85\right)\cdot E_{T}(30)\;\;\;\;\;\;\;\;\;\;R^{2}=0.95$$

Figure 9a–c collectively indicates that the solvation of the Q**1** model indicator is determined by the solvents with a strong H-bond donor and donor-accepting properties, and to a lesser extent, by the solvents with high polarity. The solvation of Q**1** is given mainly by the hydrogen bonds established with the solvent, with the contribution from the ion–dipole interactions (giving the charge-separated structure) and van der Waals forces. The fact that Q**1** is thermodynamically more stable in 1-octanol than in propanoic acid or methanol can be connected to its hydrophobic structure. The solubility is the lowest in benzene, chloroform, and 1,2-dichloroethane, due to the lack of specific intermolecular interactions.

Although IEF-PCM is useful for assessing the negative solvatochromism of Q**1**–Q**3**, it did not include the specific interactions. For this, we have constructed 1:2 complexes of the Q**1**–solvent molecule for each of the solvents used, including the non-coordinating ones. The solvent molecules were positioned near and around the active centers of Q**1**, namely the C=O functionalities, the N^+^ atom, and the carbanion, at the hydrogen-bonding distance. The DFT calculations have shown that stable 1:2 complexes were formed with explicit N,N-dimethylformamide, methanol, 1-octanol, propanoic acid, and propane-1,3-diol molecules. Benzene, chloroform, and 1,2-dichloroetane molecules did not interact with the Q**1** model, and no intermolecular complexes were formed.

**Figure 10:**
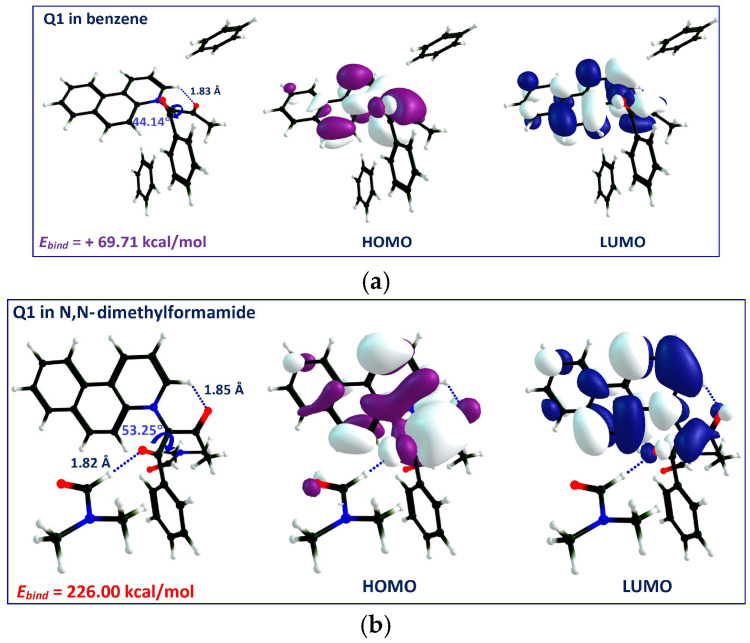
Optimized geometries of 1:2 complexes of Q**1** as the model indicator with the absolute value of the binding energy, *E_bind_*, and (**a**) benzene; (**b**) N,N-dimethylformamide (B3LYP/6-31G+(d,p)). The dotted lines mark the hydrogen bond. Color code: positive lobes—white; negative lobes—magenta (HOMO) and blue (LUMO).

Figure 10 shows the difference in DFT-optimized geometries and the HOMO–LUMO orbitals of 1:2 complexes of Q**1** between benzene (Figure 10a) and N,N-dimethylformamide (Figure 10b). The benzene molecules slipped away from the Q**1** model, and not a single contact was made between them. In contrast, one single molecule of N,N-dimethylformamide was engaged in an intermolecular hydrogen bond with Q**1**, at a 1.82 Å distance.

**Figure 11:**
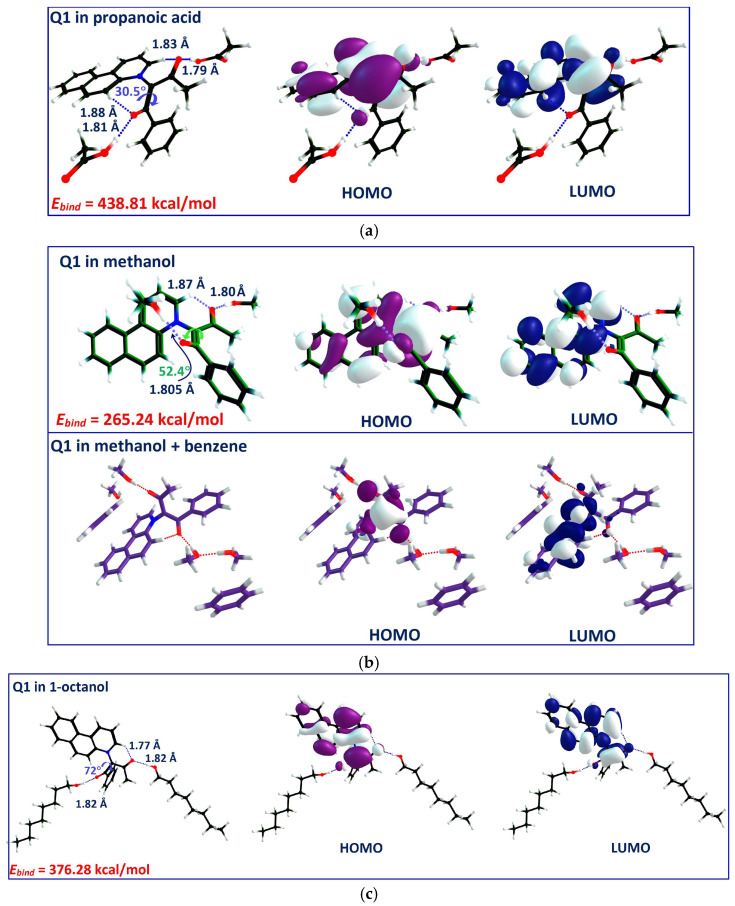

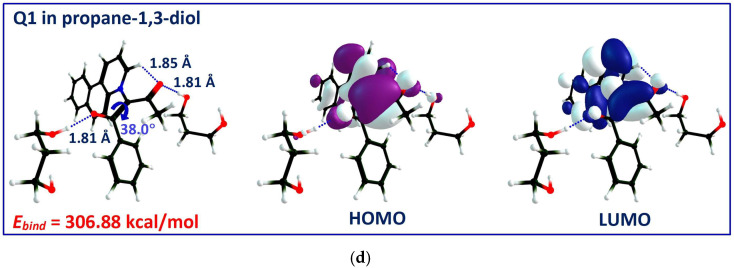
Optimized geometries of 1:2 complexes of Q**1** as the model indicator and two hydrogen bond donor (HBD) solvent molecules. HBD solvents are (presented according to their β value): (**a**) propanoic acid; (**b**) methanol; (**c**) 1-octanol; (**d**) propane-1,3-diol (B3LYP/6-31G+(d,p). The dotted lines mark the hydrogen bond. Color code: positive lobes—white; negative lobes—magenta (HOMO) and blue (LUMO).

The DFT-optimized geometries of four complexes of (Q**1**-interacting solvent) are presented in Figure 11. In contrast with N,N-dimethylformamide, Q**1** was H-bonded with methanol at both C=O groups in either methanol or the methanol + benzene solution, while the benzene molecules moved away from the proximity of Q**1** (Figure 11a). The same situation was predicted for each of the propanoic acid + chloroform and 1-octanol + 1,2-dichloroethane solutions, where the aprotic solvent did not interact with either the solute or with the co-solvent. Two intramolecular hydrogen bonds between the two C=O groups and aromatic hydrogens were formed in every case. In either solvent, the intermolecular H-bonding maintained the torsion in the N^+^-C^−^-C(=O) framework, with dihedral angles varying from 30° in propanoic acid to 72° in 1-octanol. The intermolecular H-bonds were around 1.81–1.82 Å in length, regardless of the solvent.

Following the HOMO and LUMO representations in Figure 10 and Figure 11, the charge transfer process occurs in Q**1**, regardless of what the H-bonding partner is. However, in benzene, both HOMO and LUMO are more localized around the carbanion and positive nitrogen, respectively, in comparison with the H-bonded complexes. In the 1:2 complexes, both frontier orbitals are extended and partially overlap.

Based on the simulations, the binding energy due to the (Q**1**–solvent) complex formation can be estimated according to the following equation [32]:

(24) $$E_{bind}=E_{Q1-solv}-(E_{Q1}+E_{solv})$$

where $E_{Q1-solv}$ is the total energy of (Q**1**–solvent) formation, *E_Q_*_1_ and *E_solv_* are the energies of Q**1** and the solvent, respectively, calculated individually in the gas phase. The *E_bind_* values, displayed for every (Q**1**–solvent) complex in Figure 10 and Figure 11, are the highest in magnitude when binding with propanoic acid (*E_bind_* = −438.81 kcal/mol), followed by 1-octanol. As for (Q**1**–benzene) solution, the binding energy was estimated with a positive value of +69.71 kcal/mol, indicating that this complex cannot be formed.

**Figure 12:**
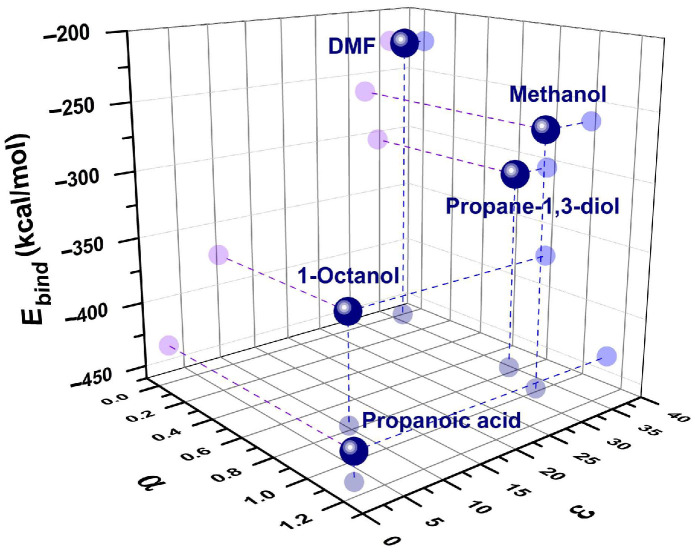
Correlation of *E_bind_* of 1:2 (Q**1**–solvent) complexes with the α and ε parameters of the coordinating solvents. DMF stands for N,N-dimethylformamide.

When plotted against the HB donor ability, α, and the dielectric permittivity of the solvents (Figure 12), this results in only the specific intermolecular interactions contributing to the stability of (Q**1**–solvent) complexes. On the contrary, the more polar the surrounding medium is, the less stable is the (Q**1**–solvent) complex. Following the above results, the selective solvation of Q**1**–Q**3** in binary mixtures is then confirmed by the formation of strong intermolecular complexes with the protic solvent.

## 3. Materials and Methods

The synthesis and structural characterization data of benzo-[f]-quinolinium acetyl benzoyl methylid (Q**1**), benzo-[f]-quinolinium carbethoxy benzoyl methylid (Q**2**), and benzo-[f]-quinolinium dibenzoyl methylid (Q**3**) were reported in detail in a previous study [7].

The spectrophotometric-grade solvents used were as follows: benzene, chloroform, 1-octano, and methanol (from Merck, KGaA, Darmstadt, Germany); N,N-dimethylformamide, propanoic acid, and propane-1,3-diol (from Sigma-Aldrich, St. Louis, MO, USA); and 1,2-dichloroethane (Chemopar, Iasi, Romania).

The electronic absorption spectra were recorded with the Analytic Jena Specord Plus-5 UV–Vis spectrophotometer (Analitik Jena GmbH+Co.KG, Jena, Germany), in quartz cuvettes with a 10 mm pathlength. The binary mixtures of a certain composition were prepared by volume. The concentration of the solute was maintained around 5 × 10^−5^ M, keeping the solution under the validity of the Lambert–Beer law. The measurements were made at ambient temperature (22° ± 2 °C) for both neat solvents and binary mixtures, at a resolution of 0.2 nm, and an uncertainty of ±0.5 nm. Each reported value is the average of duplicate experiments.

The theoretical investigations were carried out using the Gaussian 09W version A.01 package [33] (Gaussian Inc., Wallingford, CT, USA). The relaxed geometries of individual molecules and of complexes were first calculated in the gas phase and in the presence of an implicit solvent without symmetry constraints, using the PM3MM method. The gradient-corrected DFT with Becke’s three-parameter hybrid exchange functional and the Lee−Yang−Parr correlation functional (B3LYP) [34] with the 6-31G+(d,p) basis set was afterwards used for refining the geometries. In every case, the minimum on the potential energy surface was verified by calculating the harmonic vibrational frequencies. The effects of electron correlation and long-range correction on the optical properties were taken into consideration [35]. The solvents’ effects were calculated based on the optimized geometry in the gas phase by means of the implicit solvent polarizable continuum model (PCM) within the self-consistent reaction field formalism (SCRF) [36,37]. The vertical excitation energies were calculated by using TD-DFT [38,39] with the Coulomb-attenuated B3LYP functional (CAM-B3LYP/6-31G+(d,p)/IEF-PCM) and def2SV fitting model [38,39].

## 4. Conclusions

In the present work, we have investigated the behavior of three zwitterionic benzo-[f]-quinolinium ylid derivatives with intramolecular charge transfer (ICT) in four binary solvent mixtures via UV–Vis absorption spectroscopy. The Kamlet–Abboud–Taft linear solvation energy relationship revealed that the absorption maximum of the ICT band depends on specific intermolecular interactions based on the hydrogen bond acidity and basicity of the solvents, and much less on the polarity/polarizability. Within the frameworks of the Bagchi–Chatterjee solvation model and solvent exchange theory in the Bosch–Rosés model, the solutions behave like non-ideal mixtures, but with no synergistic behavior. This showed the absence of significant solvent–solvent interactions and that Q**1**–Q**3** are preferentially solvated by the polar protic solvent throughout the mixtures with the non-coordinating solvent. The Bosch–Rosés model in the general approach fits the behavior of the molar transition energy of solutes well and allows the estimation of the composition of the solvation microsphere. The application of the statistical cell model of ternary solutions leads to the calculation of the differences in energy between the solute–solvent pairs in the first solvation shell. This model neglects the interactions between the solvents, so it was successfully applied to the binary mixtures under study because the solvents do not interact with each other. The quantum chemical calculations with implicit and explicit solvents confirmed that the most important interactions explaining the solvatochromic behavior are the solute–solvent interactions based mainly on hydrogen bonding, with the solvents as HB donors and the oxygen atoms of the solute as acceptors.

## Figures and Tables

**Table 1:** Solvatochromic parameters of neat solvents and the experimental absorption maxima of the intramolecular charge transfer band of Q**1**–Q**3**.

Solvent	ε	*n*	α	β	π*	λ(Q1), cm^−1^	λ(Q2), cm^−1^	λ(Q3), cm^−1^
Poor hydrogen bond acceptors and poor hydrogen bond donors								
Benzene	2.27	1.5011	0	0.1	0.59	22,000	20,600	20,500
1,2-dichloroethane	10.3	1.3729	0	0.1	0.81	21,800	21,500	21,160
Poor hydrogen bond donor								
Chloroform	4.81	1.4459	0.2	0.1	0.69	21,500	21,000	21,500
Strong hydrogen bond acceptor and poor hydrogen bond donor								
N,N-dimethylformamide	36.71	1.4305	0	0.69	0.88	22,500	22,170	22,000
Strong hydrogen bond donors and strong hydrogen bond acceptors								
Propanoic acid	3.1	1.386	1.12	0.45	--	24,300	24,170	24,640
Methanol	32.62	1.3314	0.98	0.66	0.6	24,000	24,200	24,640
1-octanol	10.3	1.429	0.77	0.81	0.4	22,800	23,460	23,000
Propane-1,3-diol	35	1.4398	0.76	0.84	0.48	24,300	24,170	24,640

α, β, π*, ε, and *n* were taken from refs. [7,18,19].

**Table 2:** The solvation parameters from Equation (15) in the solvent mixtures.

Compound	*E_T_*_1_ (kcal/mol)	*E_T_*_2_ (kcal/mol)	*E_T_*_12_ (kcal/mol)	*f*_2/1_	*f*_12/1_	*f*_12/2_	RSS ^a^	SD ^b^
	Methanol + benzene							
Q**1**	68.6	63.0	68.5	5.2	9.5	1.8	0.99	0.10
Q**2**	69.2	58. 9	68.5	1.2	2.4	1.9	0.99	0.06
Q**3**	58.6	70.4	70.5	0.6	3.9	6.1	0.99	0.13
	Propane-1,3-diol + N,N-dimethylformamide							
Q**1**	69.5	64.3	69.3	0.5	2.4	5.1	0.99	0.10
Q**2**	69.1	63.3	67.1	0.1	0.2	3.7	0.99	0.03
Q**3**	70.4	63.8	69.3	1.2	8.9	7.4	0.98	0.35
	Propionic acid + chloroform							
Q**1**	69.5	61.4	65.5	0.05	0.13	2.60	0.99	0.05
Q**2**	68.8	60.0	64.3	0.15	0.16	1.06	0.96	0.74
Q**3**	70.4	61.4	63.3	0.02	0.21	10.5	0.99	0.03
	1-octanol + 1,2-dichloroethane							
Q**1**	65.2	62.3	65.1	0.50	5.92	11.84	1	0.008
Q**2**	67.1	61.4	58.2	0.008	0.08	10	0.99	0.27
Q**3**	65.8	60.5	61.5	0.01	0.08	8	1	0.02

^a^ RSS = residual sum of squares; ^b^ SD = standard deviation.

**Table 3:** The interaction energies in the pairs between Q**1**–Q**3** and binary solvents.

	Intercept	ω_2_ − ω_1_, × 10^21^ J
MeOH_Benzene		
Q**1**	0.73	3.00
Q**2**	0.97	3.99
Q**3**	1.18	4.85
Propane-1,3-diol + DMF		
Q**1**	1.76	7.26
Q**2**	1.86	7.64
Q**3**	1.22	5.04
Propionic acid + chloroform		
Q**1**	2.56	10.55
Q**2**	1.77	7.29
Q**3**	2.10	8.65
1-octanol + 1,2 dichloroethane		
Q**1**	2.05	8.42
Q**2**	2.37	9.74
Q**3**	2.00	8.23

**Table 4:** Calculated values, in eV, of the ionization potential—IP; electron affinity—EA; electronegativity—χ; chemical potential—μ; chemical hardness—η; electrophilicity—ω; and Mulliken charges (in e) on the N^+^ and C^−^ atoms of Q**1** in implicit solvents.

Medium	Mulliken Charge (e)		IP	EA	χ	μ	η	ω
	N^+^	C^−^						
Gas phase	0.909	−0.752	5.54	2.84	5.54	−5.54	2.84	3.25
Benzene	0.9	−0.782	5.45	2.19	5.45	−5.45	2.19	2.24
Propanoic acid	0.885	−0.777	5.53	2.34	5.53	−5.53	2.33	2.42
Chloroform	0.835	−0.770	5.54	2.21	5.54	−5.54	2.21	2.25
1,2-dichloroethane	0.839	−0.775	5.55	2.50	5.55	−5.55	2.50	2.66
1-octanol	0.908	−0.749	5.62	2.32	5.62	−5.62	2.32	2.38
Methanol	0.831	-0.767	5.64	2.47	5.64	−5.64	2.47	2.59
Propane-1,3-diol	0.894	−0.785	5.80	2.32	5.80	−5.80	2.32	2.38
N,N-dimethylformamide	0.824	−0.777	5.80	2.47	5.80	−5.80	2.47	2.57

## Data Availability

The original contributions presented in this study are included in the article/Supplementary Materials. Further inquiries can be directed to the corresponding author.

## Supplementary Materials

The following supporting information can be downloaded at https://www.mdpi.com/article/10.3390/molecules31020290/s1: Table S1. Q**1** in the binary solvent methanol (1) + benzene (2) (ε = 32.62, n = 1.3314 + ε = 2.27, n = 1.5011): percent concentration (*C*_1_, %), methanol molar fraction (*x*_1_, %), the average statistic weight of MeOH in the first solvation sphere of Q**1** (*p*_1_, %) and the wavenumber of the ICT band (ν*_t_*, cm^−1^); Table S2. Q**2** in the binary solvent methanol (1) + benzene (2) (ε = 32.62, n = 1.3314 + ε = 2.27, n = 1.5011): percent concentration (*C*_1_, %), methanol molar fraction (*x*_1_, %), the average statistic weight of MeOH in the first solvation sphere of Q**2** (*p*_1_, %) and the wavenumber of the ICT band (ν*_t_*, cm^−1^). Table S3. Q**3** in the binary solvent methanol (1) + benzene (2): percent concentration (*C*_1_, %), MeOH molar fraction (*x*_1_, %), the average statistic weight of methanol in the first solvation sphere of Q**1** (*p*_1_, %) and the wavenumber of the ICT band (ν*_t_*, cm^−1^). Table S4. Q**1**, Q**2** and Q**3** in the binary solvent propanoic acid (1) + chloroform (2) (ε = 3.10, n = 1.386 + ε = 4.81, n = 1.4459): percent concentration (*C*_1_, %), molar fraction of propanoic acid (*x*_1_, %), the average statistic weight of propanoic acid in the first solvation sphere of Q**1** (*p*_1_, %) and the wavenumber of the ICT band (ν*_t_*, cm^−1^). Table S5. Propionic acid + Chloroform (continuation of Table S4). Table S6. Q**1**, Q**2** and Q**3** in the binary solvent 1-octanol + 1,2-dichloroethane (ε = 10.3, n = 1.1429 + ε = 10.3, n = 1.3729): percent concentration (*C*_1_, %), molar fraction of 1-octanol (*x*_1_, %), the average statistic weight of 1-octanol in the first solvation sphere of Q**1** (*p*_1_, %) and the wavenumber of the ICT band (ν*_t_*, cm^−1^). Table S7. 1-Octanol + 1,2-dichloroethane (continuation of Table S6). Table S8. Q**1**, Q**2** and Q**3** in the binary solvent propane-1,3-diol and N,N-Dimethylformamide (ε = 35.0, n = 1.4398 + ε = 36.71, n = 1.4305: percent concentration (*C*_1_, %), molar fraction of 1 propane-1,3-diol (*x*_1_, %), the average statistic weight of propane-1,3-diol in the first solvation sphere of Q**1** (*p*_1_, %) and the wavenumber of the ICT band (ν*_t_*, cm^−1^). Table S9. Propane-1,3-diol + N,N-Dimethylformamide (continuation of Table S8). Table S10. Calculated energy and the dipole moments of Q**1** model in implicit solvents (B3LYP/6-31G+(d,p)/IEF-PCM level of theory). Figure S1. Plots of the local mole fraction $x_{1}^{s}$ vs. x_1_ for the polar component using Q**1**–Q**3** as indicators in: (a) Methanol + benzene; (b) Propane-1,3-diol + N,N-dimethylformamide; (c) 1-Octanol + 1,2-dichloroethane; (d) Propanoic acid + chloroform. The dotted line corresponds to the behavior of an ideal binary mixture. Figure S2. Contour plots for the HOMO and LUMO orbitals, and the electrostatic potential maps (ESP) of Q**1** in the eight studied solvents as follows: (a) in gas phase and aprotic solvents; (b) dipolar solvents. Lists with the coordinates of the optimized geometries of the 1:2 Q**1**–solvent complexes.
