# Supplementary material for: Preferential Solvation of Zwitterionic Benzo-[f]-Quinolinium Ylids in Binary Solvent Mixtures: Spectral Study and Quantum Chemical Calculations
Source: Molecules. 2026 Jan 13;31(2):290. doi: 10.3390/molecules31020290 (PMC12844157; doi:10.3390/molecules31020290)
Supplement: Supplementary file 1 [file molecules-31-00290-s001.zip › molecules-4052709-supplementary.pdf]

Supplementary Materials

Preferential Solvation of Zwitterionic Benzo-[f]-Quinolinium Ylids in Binary Solvent Mixtures: Spectral Study and Quantum Chemical Calculations

Mihaela Iuliana Avadanei [1,*], Ovidiu Gabriel Avadanei [2] and Dana Ortansa Dorohoi [2]

[1] Petru Poni Institute of Macromolecular Chemistry, 41A Gr. Ghica Voda Alley, 700487 Iasi, Romania
[2] Faculty of Physics, Alexandru Ioan Cuza University, 11 Carol I Blvd, 700506 Iasi, Romania; minu@uaic.ro (O.G.A.); ddorohoi@uaic.ro (D.O.D.)
* Correspondence: mavadanei@icmpp.ro

Table of content

S1. Experimental data 1

S2. Coordinates of the optimized geometries of the 1:2 Q1 – solvent complexes,

at the B3LYP/6-31G+(d,p) level of theory 5

Table S1. Q1 in the binary solvent methanol (1) + benzene (2) (ε = 32.62, n = 1.3314 + ε = 2.27, n = 1.5011): percent concentration (C_1, %), methanol molar fraction (x_1, %), the average statistic weight of MeOH in the first solvation sphere of Q1 (p_1, %) and the wavenumber of the ICT band (ν_t, cm^{-1})

C_1(%)	x_1(%)	$ln\frac{x_1}{1-x_1}$	$\nu\ (cm^{-1})$	p_1 (%)	$ln\frac{p_1}{1-p_1}$
**0**	0	-	22000	-	-
**5**	0.103	-2.164	22450	0.225	-1.237
**10**	0.195	-1.418	22630	0.315	-0.777
**20**	0.353	-0.606	22970	0.485	-0.060
**30**	0.483	-0.068	23275	0.638	0.567
**40**	0.593	0.376	23485	0.743	1.062
**50**	0.686	0.781	23650	0.825	1.551
**60**	0.766	1.186	23755	0.878	1.974
**70**	0.836	1.629	23840	0.920	2.443
**80**	0.897	2.164	23905	0.953	3.009
**90**	0.950	2.987	23950	0.975	3.663
**100**	1.000	-	24000		

Table S2. Q2 in the binary solvent methanol (1) + benzene (2) (ε = 32.62, n = 1.3314 + ε = 2.27, n = 1.5011): percent concentration (C_1, %), methanol molar fraction (x_1, %), the average statistic weight of MeOH in the first solvation sphere of Q2 (p_1, %) and the wavenumber of the ICT band (ν_t, cm^{-1})

C_1(%)	x_1(%)	$ln\frac{x_1}{1-x_1}$	$\nu\ (cm^{-1})$	p_1 (%)	$ln\frac{p_1}{1-p_1}$
**0**	0	-	20600	-	-
**5**	0.103	-2.164	21275	0.188	-1.463
**10**	0.195	-1.418	21800	0.333	-0.695
**20**	0.353	-0.606	22590	0.553	0.213
**30**	0.483	-0.068	23095	0.693	0.675
**40**	0.593	0.376	23495	0.804	1.411
**50**	0.686	0.781	23720	0.867	1.875
**60**	0.766	1.186	23950	0.931	2.602
**70**	0.836	1.629	24060	0.961	3.200
**80**	0.897	2.164	24077	0.966	3.346
**90**	0.950	2.987	24140	0.983	4.057
**100**	1.000	-	24200		

Table S3. Q3 in the binary solvent methanol (1) + benzene (2): percent concentration (C_1, %), MeOH molar fraction (x_1, %), the average statistic weight of methanol in the first solvation sphere of Q1 (p_1, %) and the wavenumber of the ICT band (ν_t, cm^{-1})

C_1(%)	x_1(%)	$ln\frac{x_1}{1-x_1}$	$\nu\ (cm^{-1})$	p_1 (%)	$ln\frac{p_1}{1-p_1}$
**0**	0	-	20500	-	-
**5**	0.103	-2.164	21850	0.326	-0.726
**10**	0.195	-1.418	22470	0.476	-0096
**20**	0.353	-0.606	23265	0.668	-0.699
**30**	0.483	-0.068	23665	0.764	1.175
**40**	0.593	0.376	24035	0.850	1.766
**50**	0.686	0.781	24205	0.895	2.143
**60**	0.766	1.186	24320	0.923	2.484
**70**	0.836	1.629	24445	0.953	3.009
**80**	0.897	2.164	23905	0.969	3.442
**90**	0.950	2.987	24510	0.978	3.794
**100**	1.000	-	24640	-	-

Table S4. Q1, Q2 and Q3 in the binary solvent propanoic acid (1) + chloroform (2) (ε = 3.10, n = 1.386 + ε = 4.81, n = 1.4459): percent concentration (C_1, %), molar fraction of propanoic acid (x_1, %), the average statistic weight of propanoic acid in the first solvation sphere of Q1 (p_1, %) and the wavenumber of the ICT band (ν_t, cm^{-1})

C_1(%)	x_1	$ln\frac{x_1}{1-x_1}$	ν (cm^{-1})		
			**Q1**	**Q2**	**Q3**
**0**	0	-	21500	21000	21500
**5**	0.053	-2.883	21790	21880	22260
**10**	0.109	-2.101	22290	22400	22850
**25**	0.263	-1.-30	23360	23530	2376-
**50**	0.51-	0.040	24030	23690	24280
**75**	0.763	0.763	24260	23910	24500
**100**	1.000	-	24300	24170	24640

Table S5. Propionic acid + Chloroform (continuation of Table S4).

p_1 (%)			$ln\frac{p_1}{1-p_1}$		
**Q1**	**Q2**	**Q3**	**Q1**	**Q2**	**Q3**
**-**	-	-	-	-	-
**0.104**	0.278	0.242	-2.154	-0.954	-1.142
**0.264**	0.442	0.430	-1.025	-0.233	-0.282
**0.664**	0.754	0.720	0.681	1.12	0.944
**0.904**	0.817	0.885	2.242	1.719	2.040
**0.986**	0.918	0.955	4.254	2.415	3.055
**1**	1	1	-	-	-

Table S6. Q1, Q2 and Q3 in the binary solvent 1-octanol + 1,2-dichloroethane (ε = 10.3, n = 1.1429 + ε = 10.3, n = 1.3729): percent concentration (C_1, %), molar fraction of 1-octanol (x_1, %), the average statistic weight of 1-octanol in the first solvation sphere of Q1 (p_1, %) and the wavenumber of the ICT band (ν_t, cm^{-1})

C_1(%)	x_1	$ln\frac{x_1}{1-x_1}$	ν (cm^{-1})		
			**Q1**	**Q2**	**Q3**
**0**	0	-	21800	21500	21160
**5**	0.025	-3.664	22050	21700	21300
**10**	0.052	-2.903	22220	21900	21550
**25**	0.141	-1.807	22480	22940	22200
**50**	0330	-0.708	22654	23250	22720
**75**	0.597	1.169	22740	23380	22904
**100**	1.000	-	22800	23460	23000

Table S7. 1-Octanol + 1,2-dichloroethane (continuation of Table S6).

p_1 (%)			$ln\frac{p_1}{1-p_1}$		
**Q1**	**Q2**	**Q3**	**Q1**	**Q2**	**Q3**
**-**	-	-	-	-	-
**0.250**	0.102	0.076	-1.099	-2.175	-2.408
**0.420**	0.204	0.212	-0.286	-1.361	-1.313
**0.680**	0.735	0.565	0.754	1.020	0.261
**0.854**	0.893	0.848	1.766	2.122	1.719
**0.940**	0.959	0.948	2.752	3.152	2.903
**1**	1	1	-	-	-

Table S8. Q1, Q2 and Q3 in the binary solvent propane-1,3-diol and N,N-Dimethylformamide (ε = 35.0, n = 1.4398 + ε = 36.71, n = 1.4305: percent concentration (C_1, %), molar fraction of 1 propane-1,3-diol (x_1, %), the average statistic weight of propane-1,3-diol in the first solvation sphere of Q1 (p_1, %) and the wavenumber of the ICT band (ν_t, cm^{-1})

C_1(%)	x_1	$ln\frac{x_1}{1-x_1}$	$\nu\ (cm^{-1})$		
			**Q1**	**Q2**	**Q3**
**0**	0	-	22500	22170	22000
**5**	0.054	-2.883	22900	22500	22800
**10**	0.107	-2.122	23200	22800	23400
**25**	0.264	-1.025	23630	23500	23680
**50**	0.518-	0.072	24100	23960	24120
**75**	0.792	1.337	24220	24100	24310
**100**	1.000	-	24300	24170	24640

Table S9. Propane-1,3-diol + N,N-Dimethylformamide (continuation of Table S8)

p_1 (%)			$ln\frac{p_1}{1-p_1}$		
**Q1**	**Q2**	**Q3**	**Q1**	**Q2**	**Q3**
-	-	-	-	-	-
0.104	0.222	0.303	-1.254	-1.621	-0.833
0.264	0.389	0.530	-0.452	-0.777	0.120
0.664	0.628	0.636	0.524	0.686	0.558
0.904	0.889	0.803	2.081	2.143	1.405
0.986	0.956	0.875	3.079	3.317	1.946
1	1	1	-	-	-

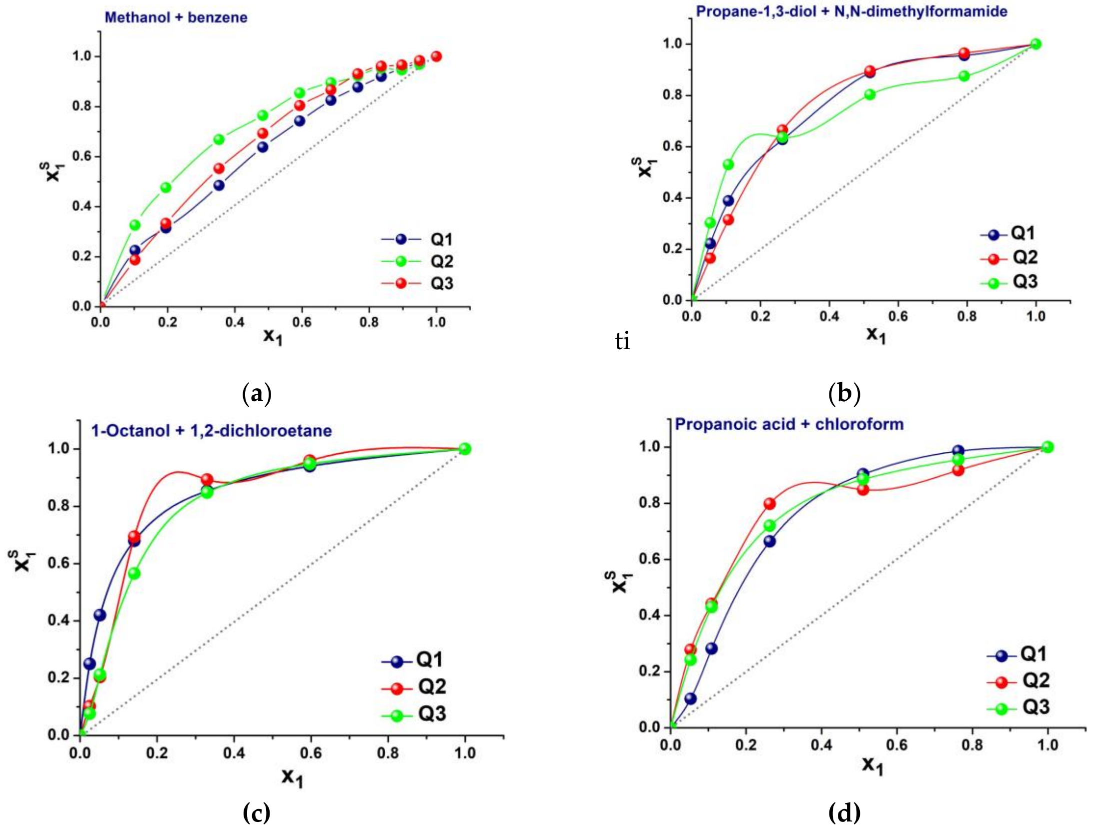

Figure S1. Plots of the local mole fraction x_1^s vs. x_1 for the polar component using Q1 – Q3 as indicators in: (**a**) Methanol + benzene; (**b**) Propane-1,3-diol + N,N-dimethylformamide; (c) 1-Octanol + 1,2-dichloroethane; (d) Propanoic acid + chloroform. The dotted line corresponds to the behavior of an ideal binary mixture.

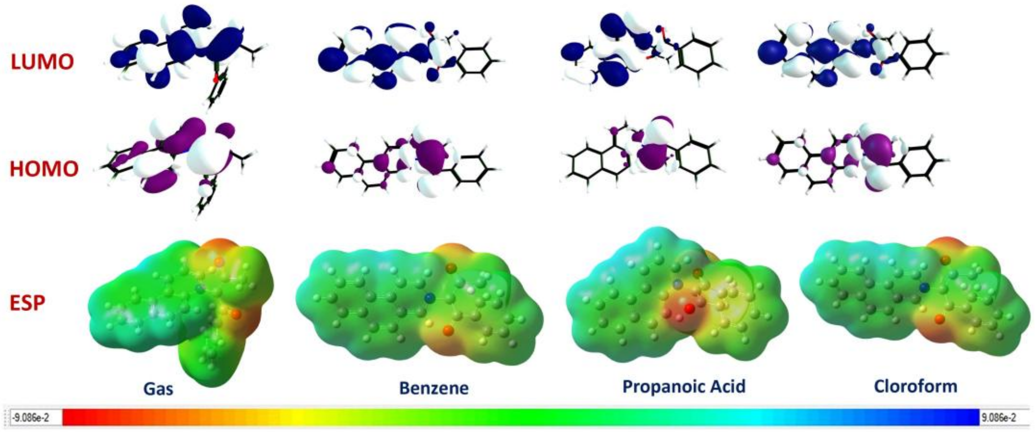

(a)

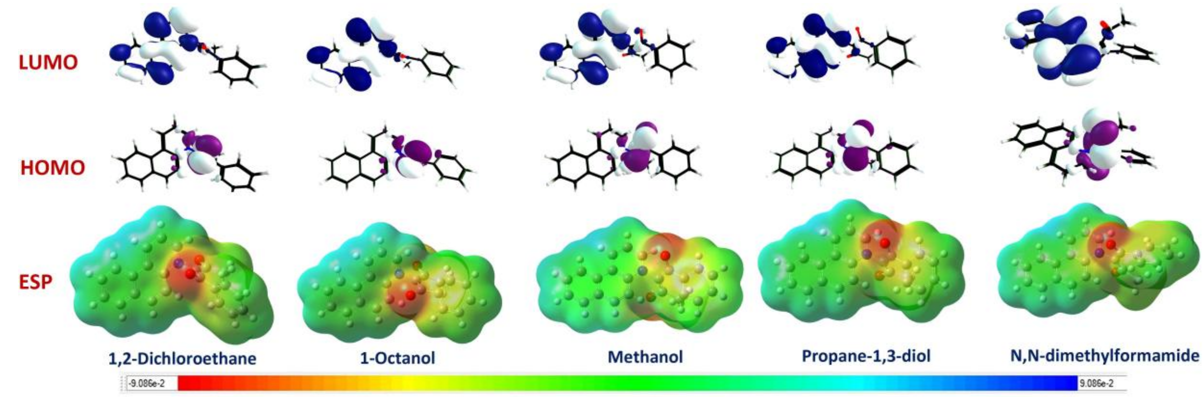

(b)

Figure S2. Contour plots for the HOMO and LUMO orbitals, and the electrostatic potential maps (ESP) of Q1 in the eight studied solvents as follows: (a) in gas phase and aprotic solvents; (b) dipolar

Table S10. Calculated energy and the dipole moments of Q1 model in implicit solvents (B3LYP/6-31G+(d,p)/IEF-PCM level of theory)

Implicit solvent	Energy (hartree)	Dipol moment (Debye)	Energy (kcal/mol)	Energy difference from gas (kcal/mol)
**Gas phase**	-1091.599781	5.2197	-684989.2329	0
**Benzene**	-1091.615146	5.861	-684998.8743	-9.641407363
**1,2-Dichlororethane**	-1091.618829	6.9016	-685001.1856	-11.95268173
**Cloroform**	-1091.62036	6.4365	-685002.1463	-12.9134427
**propanoic acid**	-1091.620795	6.037	-685002.4191	-13.1862148
**MeOH, B3L**	-1091.622586	6.5954	-685003.5428	-14.30993372
**Propane-1,3-diol**	-1091.631835	6.6435	-685009.3473	-20.11435267
**octanol**	-1091.638303	6.2505	-685013.4057	-24.17277663
**DMF, B3LYP**	-1091.635088	7.2276	-685011.3884	-22.15545282

S2. Coordinates of the optimized geometries of the 1:2 Q1 – solvent complexes, at the B3LYP/6-31G+(d,p) level of theory

1-Octanol - Q1 complex

	Coordinates (Å)		
	**X**	**Y**	**Z**
C	-2.45352359	0.87953833	0.19198597
C	-1.07871822	0.51690308	0.08003011
C	-2.79618292	2.25085715	0.23528503
N	-0.08410000	1.50160000	0.11770000
C	-1.79924592	3.21135559	0.07514721
C	-0.45268132	2.80884421	0.15797987
C	-1.69572131	-1.83013156	-0.11255972
C	-0.73537110	-0.87055451	-0.11194257
C	-3.07688366	-1.50757895	0.08203596
C	-3.46033756	-0.15503929	0.23379255
C	-5.38449026	-2.21710203	0.30774243
C	-4.05403553	-2.52884457	0.11919492

C	-5.77249244	-0.87414180	0.46614265
C	-4.83152565	0.13581048	0.43063863
C	1.43270283	1.22873673	0.22049890
C	1.62879671	-0.21767747	0.21969755
C	2.51806629	2.17721098	0.36390004
C	2.76365502	-0.78082897	-0.55735334
C	3.91612677	1.64846502	0.47474971
O	2.33002414	3.41279964	0.43360181
C	3.61134845	0.04554895	-1.30484355
C	4.62046490	-0.51319146	-2.08751118
C	4.79299832	-1.89724780	-2.12466920
C	2.93360418	-2.17066003	-0.60629195
C	3.95054441	-2.72452082	-1.38216594
O	0.85806256	-0.99213481	0.81859974
H	-3.84977862	2.56318972	0.28964006
H	-2.02700839	4.28732723	0.09321469
H	0.36350086	3.55568972	0.24653754
H	-1.42639769	-2.88827114	-0.26440417
H	0.32240285	-1.14493359	-0.27210184
H	-6.14638293	-3.01030383	0.33812947
H	-3.73449199	-3.57531444	-0.00144314
H	-6.83516426	-0.63356133	0.62174293
H	-5.16319608	1.17541357	0.56714508
H	4.34270750	1.49766382	-0.54742066
H	4.55186871	2.39194490	1.01598663
H	3.94074285	0.67767918	1.02447863
H	3.48380539	1.13890575	-1.26107627
H	5.28239264	0.13989811	-2.67491090
H	5.59212330	-2.33536187	-2.74057481
H	2.24788830	-2.81610471	-0.03482112
H	4.08362181	-3.81584933	-1.41222834
O	2.25738568	5.86148212	0.48305985
H	2.25126568	4.89902212	0.36260985
C	3.16823568	6.18927212	0.42120985
O	-1.02668153	-2.04202254	1.99295205
H	-0.20751153	-1.69524254	1.60612205
C	-1.41396153	-1.37503254	2.58117205
H	3.56971568	5.94771212	-0.54076015
H	3.76471568	5.73073212	1.18206985
H	-1.63088153	-0.48896254	2.02192205
H	-0.72089153	-1.14293254	3.36258205
C	3.17803711	7.71731719	0.61247455
H	2.76728634	7.95951213	1.57035002
H	2.58976337	8.17652596	-0.15431180
C	4.62568755	8.23586550	0.52871376
H	5.04150621	7.98043443	-0.42352036
H	5.21040606	7.78810649	1.30493288
C	4.63331246	9.76648241	0.69828302
H	4.21375461	10.02212041	1.64881993
H	4.05194442	10.21448767	-0.08030705
C	6.08152292	10.28437927	0.62037734
H	6.50123876	10.02832754	-0.32997845
H	6.66277765	9.83673570	1.39925998

C	6.08908409	11.81507187	0.78926486
H	5.66940614	12.07112179	1.73963787
H	5.50779570	12.26271325	0.01040608
C	7.53728903	12.33297527	0.71130008
H	7.95681167	12.07733361	-0.23925139
H	8.11868947	11.88497801	1.48987056
C	7.54491304	13.86359329	0.88085942
H	8.55111042	14.22346249	0.82644437
H	7.12561530	14.11922276	1.83151340
H	6.96331104	14.31157607	0.10243117
C	-2.71440306	-1.92564777	3.19539896
H	-3.02865488	-2.79036972	2.64913735
H	-3.47688481	-1.17653918	3.14684080
C	-2.46515311	-2.30986636	4.66572224
H	-2.07050594	-1.46644789	5.19278855
H	-1.76463132	-3.11748836	4.70949370
C	-3.79212822	-2.74560528	5.31446613
H	-4.19400060	-3.58005026	4.77864884
H	-4.48790824	-1.93330387	5.28353738
C	-3.53930439	-3.14947417	6.77890118
H	-3.14278174	-2.31336131	7.31610234
H	-2.83916185	-3.95800743	6.81011626
C	-4.86485910	-3.59303723	7.42524207
H	-5.26352563	-4.42647667	6.88547945
H	-5.56359717	-2.78315205	7.39783582
C	-4.61097149	-4.00269992	8.88788268
H	-4.21738517	-3.16770232	9.42896384
H	-3.90810812	-4.80899767	8.91557793
C	-5.93515224	-4.45366911	9.53191073
H	-5.75854534	-4.73939155	10.54782052
H	-6.32932085	-5.28794398	8.99013906
H	-6.63763807	-3.64700766	9.50524708

Propanoic acid - Q1 complex

	Coordinates (Å)		
	X	Y	Z
C	-1.23312000	-2.59242100	-0.05051700
C	-0.81032400	-1.31383300	-0.47377300
C	-0.27979800	-3.61113100	0.16880100
N	0.57319000	-1.02366600	-0.55891300
C	1.04856600	-3.35240100	-0.06315500
C	1.45345300	-2.05940800	-0.41672500
C	-3.13033500	-0.61929000	-0.72020800
C	-1.80939300	-0.35899400	-0.86867600
C	-3.58521100	-1.86935200	-0.19092100
C	-2.64217900	-2.86129900	0.12727000
C	-5.38182800	-3.33839400	0.48157500
C	-4.96214600	-2.12234500	-0.00590500
C	-4.44263500	-4.33692600	0.79475700

C	-3.09834100	-4.10496600	0.61967400
C	1.07794000	0.26546800	-0.76926700
C	0.35523800	1.47322800	-0.27687800
C	2.35678100	0.48846700	-1.40224800
C	0.58001200	1.86662300	1.14275200
C	2.74305900	1.90084500	-1.74834700
O	3.14171800	-0.43960100	-1.66714400
C	1.79225100	1.58472800	1.77621100
C	1.99284100	1.96120900	3.09911000
C	0.98392300	2.61516900	3.79916500
C	-0.42963900	2.52613200	1.84952200
C	-0.22706600	2.89527600	3.17373900
O	-0.40127200	2.10073900	-1.00545500
H	-0.62441600	-4.60228900	0.50168100
H	1.81273800	-4.13053100	0.04464600
H	2.53385000	-1.82950400	-0.63037400
H	-3.87192400	0.14621500	-1.00405300
H	-1.49669700	0.60667000	-1.32179300
H	-6.44902900	-3.53815400	0.62702600
H	-5.68787700	-1.34013900	-0.25760800
H	-4.79035200	-5.30192900	1.17911200
H	-2.35656600	-4.88247900	0.85916100
H	2.65194500	2.56067300	-0.87400600
H	3.77846100	1.96855500	-2.11100700
H	2.08711700	2.29497300	-2.53625100
H	2.58687600	1.06607800	1.22360100
H	2.94753100	1.74005200	3.58907100
H	1.14307500	2.91002000	4.84205100
H	-1.38882400	2.75367200	1.35602300
H	-1.02157600	3.41104900	3.72365400
C	6.44708600	-0.40103800	-1.48698800
H	7.47967700	-0.03401700	-1.53636500
H	5.77356000	0.45359000	-1.64694100
H	6.29776400	-1.10087500	-2.32004900
C	6.16537500	-1.07912400	-0.17564600
O	6.95910500	-1.35739900	0.70140500
O	4.91787000	-1.46775800	0.16338800
H	4.27597900	-1.22975000	-0.51431000
C	-3.70538500	2.85782400	-0.16958400
H	-3.74355500	1.87398100	-0.67164000
H	-4.67245300	3.01608000	0.32346700
H	-2.93114200	2.80082800	0.61477600
C	-3.40525000	3.95382200	-1.15637900
O	-4.18267200	4.78354300	-1.58435200
O	-2.17265900	4.11778400	-1.67496000
H	-1.56646700	3.44720600	-1.35469100

N,N-dimethylformamide + Q1 complex

Coordinates (Å)

	X	Y	Z
C	-2.8041	0.3204	-0.2596
C	-1.4253	0.133	-0.2119
C	-3.3933	0.7352	-1.4425
N	-0.6519	0.3768	-1.3484
C	-2.6372	0.9669	-2.5736
C	-1.2709	0.7858	-2.5216
C	-1.6189	-0.5228	2.0991
C	-0.8436	-0.293	0.9743
C	-2.9887	-0.3282	2.0517
C	-3.5863	0.0922	0.8686
C	-5.1339	-0.3592	3.1357
C	-3.7624	-0.5532	3.1806
C	-5.7334	0.0577	1.9588
C	-4.961	0.2826	0.8307
C	0.8119	0.1831	-1.3464
C	1.39469	0.30919	0.05095
C	1.4418	0.7725	-2.599
C	2.84583	0.14664	0.25546
C	2.93429	0.68004	-2.81123
O	0.7446	1.318	-3.42531
C	3.47165	0.79939	1.30659
C	4.83641	0.65649	1.49902
C	5.57492	-0.14128	0.63877
C	3.58649	-0.65177	-0.60283
C	4.9517	-0.79681	-0.41174
O	0.67072	0.53126	0.99495
H	-4.4934	0.8849	-1.4849
H	-3.1237	1.2959	-3.5167
H	-0.6634	0.9711	-3.4331
H	-1.1407	-0.8642	3.042
H	0.2549	-0.4519	1.0239
H	-5.7518	-0.5377	4.0416
H	-3.2818	-0.89	4.124
H	-6.833	0.2117	1.9196
H	-5.447	0.6178	-0.1104
H	3.2685	-0.3654	-2.63875
H	3.17917	0.97997	-3.85269
H	3.45204	1.35669	-2.09788
H	2.87874	1.43805	1.99571
H	5.33739	1.18027	2.34107
H	6.66937	-0.2557	0.79182
H	3.08534	-1.17577	-1.44463
H	5.54506	-1.43707	-1.09899
N	-1.36991	3.51275	2.29744
C	-1.61991	4.62465	3.22424
C	-2.49581	3.25845	1.38914
C	-0.22311	2.74545	2.32044
O	0.65669	2.99625	3.11234
H	-2.30561	5.36505	2.75924

H	-2.08401	4.22275	4.15134
H	-0.66491	5.13155	3.48014
H	-2.37991	2.26525	0.90374
H	-2.51361	4.05115	0.60964
H	-3.45411	3.27125	1.95214
H	0.00419	1.87765	1.66514
N	-1.14029	-3.28157	-1.65229
C	-1.08453	-4.65609	-2.16762
C	-2.45007	-2.9336	-1.08593
C	-0.09201	-2.38932	-1.7484
O	0.95364	-2.7361	-2.24905
H	-1.779	-5.30865	-1.59609
H	-1.38065	-4.65074	-3.23934
H	-0.05355	-5.05899	-2.0722
H	-2.53276	-1.83238	-0.95984
H	-2.55503	-3.4281	-0.0955
H	-3.26398	-3.27823	-1.75996
H	-0.0899	-1.33305	-1.40379

Propane-1,3-diol + Q1 complex

	Coordinates (Å)		
	**X**	**Y**	**Z**
C	0.3819	3.3894	0.2271
C	0.4106	1.9744	0.181
C	-0.8481	4.0771	0.275
N	-0.8191	1.251	0.0429
C	-2.0124	3.3619	0.291
C	-1.9723	1.9698	0.1723
C	2.838	2.0496	0.4242
C	1.6969	1.338	0.258
C	2.8356	3.477	0.3564
C	1.6098	4.1488	0.2613
C	4.0217	5.5798	0.3259
C	4.044	4.2072	0.3891
C	2.7953	6.2602	0.2306
C	1.6131	5.5604	0.1995
C	-0.932	-0.1348	-0.1502
C	0.17663	-1.07398	-0.43962
C	-2.25759	-0.78506	-0.22106
C	0.45752	-2.12809	0.60755
C	-2.40195	-2.28554	-0.38993
O	-3.30161	-0.12098	-0.19411
C	0.00424	-1.99639	1.93351
C	0.36075	-2.94081	2.8873
C	1.1442	-4.02831	2.52947
C	1.30755	-3.18908	0.28186
C	1.63842	-4.14593	1.23537
O	0.87012	-1.01993	-1.44267
H	-0.8576	5.1756	0.3246
H	-2.9886	3.8532	0.3687

H	-2.9528	1.4225	0.1274
H	3.8001	1.5366	0.5573
H	1.7595	0.2378	0.2639
H	4.9552	6.1517	0.3487
H	4.9934	3.6634	0.4613
H	2.7893	7.354	0.1788
H	0.6567	6.0975	0.1196
H	-2.37125	-2.75709	0.57007
H	-1.60034	-2.65516	-0.99466
H	-0.60553	-1.137	2.24306
H	0.04456	-2.81661	3.92795
H	1.17627	-4.93068	3.1422
H	1.71356	-3.26811	-0.73618
H	2.29585	-4.97728	0.95749
C	-7.22747	-0.96036	1.60366
H	-8.07927	-0.26923	1.76122
O	-7.1464	-1.34339	0.25869
H	-7.46676	-1.91093	2.11231
C	-5.93198	-0.35795	2.14997
C	-5.7125	1.09412	1.72691
H	-5.0651	-0.98253	1.85308
H	-5.96744	-0.39742	3.25572
O	-5.41136	1.25256	0.36537
H	-4.91668	1.55674	2.34295
H	-6.62668	1.70274	1.847
H	-6.98123	-0.55934	-0.25144
H	-4.62853	0.74651	0.18493
C	-0.704	-1.06846	-5.60197
H	-0.11283	-1.0548	-6.53919
O	-0.76054	-2.36476	-5.0744
H	-1.75753	-0.86794	-5.86549
C	-0.17845	-0.02551	-4.61414
C	1.34137	-0.05102	-4.45609
H	-0.67187	-0.14959	-3.62885
H	-0.47467	0.97827	-4.97526
O	1.82599	-1.18205	-3.78119
H	1.68913	0.86783	-3.94493
H	1.85772	-0.10895	-5.43106
H	0.12768	-2.62426	-4.86017
H	1.41646	-1.20138	-2.92479
H	-3.3361	-2.50283	-0.86433

MeOH + Q1 complex

	Coordinates (Å)		
	**X**	**Y**	**Z**
C	-2.45352	0.87954	0.19199
C	-1.07872	0.5169	0.08003
C	-2.79618	2.25086	0.23529
N	-0.0841	1.5016	0.1177
C	-1.79925	3.21136	0.07515
C	-0.45268	2.80884	0.15798
C	-1.69572	-1.83013	-0.11256
C	-0.73537	-0.87055	-0.11194
C	-3.07688	-1.50758	0.08204
C	-3.46034	-0.15504	0.23379
C	-5.38449	-2.2171	0.30774
C	-4.05404	-2.52884	0.11919
C	-5.77249	-0.87414	0.46614
C	-4.83153	0.13581	0.43064
C	1.4327	1.22874	0.2205
C	1.6288	-0.21768	0.2197
C	2.51807	2.17721	0.3639
C	2.76366	-0.78083	-0.55735
C	3.91613	1.64847	0.47475
O	2.33002	3.4128	0.4336
C	3.61135	0.04555	-1.30484
C	4.62046	-0.51319	-2.08751
C	4.793	-1.89725	-2.12467
C	2.9336	-2.17066	-0.60629
C	3.95054	-2.72452	-1.38217
O	0.85806	-0.99213	0.8186
H	-3.84978	2.56319	0.28964
H	-2.02701	4.28733	0.09321
H	0.3635	3.55569	0.24654
H	-1.4264	-2.88827	-0.2644
H	0.3224	-1.14493	-0.2721
H	-6.14638	-3.0103	0.33813
H	-3.73449	-3.57531	-0.00144
H	-6.83516	-0.63356	0.62174
H	-5.1632	1.17541	0.56715
H	4.34271	1.49766	-0.54742
H	4.55187	2.39194	1.01599
H	3.94074	0.67768	1.02448
H	3.48381	1.13891	-1.26108
H	5.28239	0.1399	-2.67491
H	5.59212	-2.33536	-2.74057
H	2.24789	-2.8161	-0.03482
H	4.08362	-3.81585	-1.41223
O	2.25739	5.86148	0.48306
H	2.25127	4.89902	0.36261
C	3.16824	6.18927	0.42121
O	-1.02668	-2.04202	1.99295
H	-0.20751	-1.69524	1.60612
C	-1.41396	-1.37503	2.58117

H	3.17505	7.25095	0.5541
H	3.56972	5.94771	-0.54076
H	3.76472	5.73073	1.18207
H	-2.31756	-1.75762	3.00796
H	-1.63088	-0.48896	2.02192
H	-0.72089	-1.14293	3.36258

Benzene + Q1 non-complex

	Coordinates (Å)		
	**X**	**Y**	**Z**
C	0.16602	2.82006	-0.01273
C	0.27731	1.43304	-0.24434
C	-0.92293	3.32081	0.73418
N	-0.76024	0.56409	0.16801
C	-1.83866	2.44247	1.25821
C	-1.74376	1.07787	0.96449
C	2.45869	1.76053	-1.27056
C	1.48938	0.9246	-0.82641
C	2.31479	3.18288	-1.16256
C	1.18708	3.71289	-0.51255
C	3.16451	5.41438	-1.53523
C	3.30143	4.05264	-1.67628
C	2.04451	5.94814	-0.87402
C	1.07383	5.11408	-0.37007
C	-0.78608	-0.78863	-0.20744
C	-0.47977	-1.1503	-1.61216
C	-1.4328	-1.76869	0.65782
C	0.39444	-2.3369	-1.87974
C	-1.40947	-3.22166	0.26991
O	-2.00873	-1.4317	1.69804
C	1.5225	-2.55105	-1.08903
C	2.37537	-3.61594	-1.34757
C	2.09895	-4.48288	-2.39998
C	0.12358	-3.20313	-2.93957
C	0.97446	-4.27509	-3.19231
O	-0.9378	-0.49756	-2.53882
H	-1.00073	4.40584	0.9008
H	-2.66912	2.78438	1.88628
H	-2.44904	0.32939	1.41679
H	3.38069	1.36143	-1.71065
H	1.65168	-0.1692	-0.86591
H	3.92668	6.09153	-1.93536
H	4.17356	3.6287	-2.18749
H	1.95004	7.03376	-0.76477
H	0.19267	5.52144	0.14907
H	-1.76406	-3.36294	-0.76017
H	-0.38327	-3.61243	0.32503
H	-2.03803	-3.83562	0.92848
H	1.72811	-1.84593	-0.25977
H	3.26409	-3.75668	-0.71137
H	2.76666	-5.32593	-2.60526

H	-0.75521	-3.03229	-3.57247
H	0.75821	-4.9557	-4.02253
H	6.80912	-1.81518	3.74091
C	5.8716	-1.73071	3.18184
H	5.15513	-0.06459	4.34926
H	6.33118	-3.38574	1.87329
C	4.94604	-0.74964	3.52138
C	5.60652	-2.60733	2.13372
C	3.75546	-0.64102	2.80883
C	4.41728	-2.49549	1.42077
C	3.49512	-1.50742	1.75144
H	3.0301	0.14162	3.05391
H	4.19517	-3.19409	0.59951
H	2.56325	-1.41805	1.17213
H	-6.18372	0.33378	3.14879
H	-8.19501	-0.94916	2.48262
C	-6.37397	0.13974	2.08952
C	-7.50904	-0.57473	1.71578
H	-4.59232	1.15709	1.40676
C	-5.4955	0.60517	1.11597
C	-7.77499	-0.81177	0.37076
C	-5.75306	0.35156	-0.22761
H	-8.67617	-1.36053	0.07925
C	-6.89386	-0.35066	-0.60085
H	-5.04979	0.69515	-0.98863
H	-7.09576	-0.54154	-1.65982
